# Metabolic Syndrome Indicators and Cardiovascular/Endocrine Risks in Rural Ecuador: A Cross-Sectional Study

**DOI:** 10.3390/jpm15030078

**Published:** 2025-02-20

**Authors:** Guillermo Fernando León-Samaniego, Holguer Estuardo Romero Urréa, Freddy Espinoza-Carrasco, Mariana de Jesús Llimaico Noriega, Grecia Elizabeth Encalada Campos, Pedro Herrera, Angela Chavez-Cembellin, Marco Faytong-Haro

**Affiliations:** 1 Facultad de Salud y Servicios Sociales, Universidad Estatal de Milagro, Milagro 091050, Ecuador; hromerou@unemi.edu.ec (H.E.R.U.); fespinozac@unemi.edu.ec (F.E.-C.); mllimaicon@unemi.edu.ec (M.d.J.L.N.); pherreram2@unemi.eduec (P.H.); 2 Facultad de Ciencias de la Salud, Universidad Espíritu Santo, Samborondón 0901962, Ecuador; angela.chavez@uees.edu.ec; 3 Facultad de Investigación, Universidad Estatal de Milagro, Milagro 091050, Ecuador

**Keywords:** metabolic syndrome (MetS), cardiovascular diseases (CVDs), endocrine diseases, hyperglycemia, dyslipidemia, rural population, Ecuador, cross-sectional study, logistic regression, public health interventions

## Abstract

**Background/Objectives:** This study assessed the prevalence of metabolic syndrome (MetS) and its association with cardiovascular and endocrine diseases in a rural Ecuadorian parish population. **Methods:** This cross-sectional study included 200 participants. Descriptive statistics were computed for glucose, total cholesterol, and triglyceride levels. Logistic regression estimated the odds ratios (ORs) for the likelihood of cardiovascular (hypertension, coronary artery disease, stroke) and endocrine diseases (diabetes and other metabolic disorders) in relation to MetS biomarkers. **Results:** The study included 200 participants, with average glucose (123.09 mg/dL), cholesterol (229.58 mg/dL), and triglycerides (188.75 mg/dL) levels exceeding standard thresholds. Logistic regression analysis showed that glucose was the strongest predictor, increasing cardiovascular disease odds by 6.9% (OR = 1.069, *p* < 0.001) and endocrine disease odds by 11.8% (OR = 1.118, *p* < 0.001) after adjustment. Cholesterol and triglycerides also significantly contributed to the risk of both diseases. The models demonstrated a high predictive performance (AUC: 0.933 for cardiovascular disease and 0.993 for endocrine diseases). **Conclusions:** MetS was significantly associated with cardiovascular and endocrine disease risks in the rural population. Integrating personalized healthcare, such as tailored dietary counseling, culturally adapted interventions, and mobile health technologies, is crucial for improving the early detection and management of MetS in underserved communities.

## 1. Introduction

Metabolic syndrome (MetS) refers to a group of conditions that increase the risk of cardiovascular disease (CVD), type 2 diabetes mellitus (T2DM), and other chronic diseases [1]. These conditions include central obesity, insulin resistance, atherogenic dyslipidemia, and hypertension, which together exacerbate the risk of developing more severe health complications [2]. The global prevalence of MetS has increased significantly over the past few decades, fueled by an increase in sedentary lifestyles, unhealthy diets, and a consequent rise in obesity rates [3]. The National Cholesterol Education Program Adult Treatment Panel III (NCEP ATP III) defines MetS based on the presence of three or more of the following factors: abdominal obesity, high triglycerides, low HDL cholesterol, high blood pressure, and elevated fasting glucose [4].

The relevance of MetS lies not only in its ability to predict cardiovascular outcomes but also in its significant relationship with endocrine diseases, particularly type 2 diabetes and metabolic disorders [5]. Moreover, studies have suggested that the presence of MetS may lead to increased risks of kidney disease, polycystic ovary syndrome, and non-alcoholic fatty liver disease [6,7]. Additionally, evidence has shown a correlation between MetS and mortality related to cardiovascular diseases, particularly coronary artery disease and stroke [8].

Globally, MetS affects approximately 25% of the adult population, and its prevalence varies significantly by region. For instance, studies have shown that in the United States, between 2011 and 2016, approximately 34.7% of adults had MetS [9]. Similar trends have been observed in other parts of the world, such as Latin America, where increasing rates of MetS contribute to the growing burden of noncommunicable diseases (NCDs) [10,11].

Despite growing concerns about the increase in non-communicable diseases in Ecuador, limited data are available regarding the prevalence of MetS, particularly in rural areas where access to healthcare is limited. Rural populations face significant barriers to healthcare, including geographical isolation, limited healthcare resources, and lower awareness of chronic disease risk factors. Previous studies in rural Latin American populations have demonstrated the effectiveness of community-based screening programs, culturally tailored health education, and mobile health initiatives in addressing MetS risk factors [12]. For example, community-led metabolic screening projects in Mexico and Brazil have improved early detection and lifestyle modification outcomes [13,14]. Similarly, mobile health applications have been successfully used in remote Andean communities to promote diabetes management and reduce cardiovascular risks. However, Ecuador lacks a comprehensive assessment of these approaches, which limits their application in rural settings. Therefore, understanding the prevalence of MetS in rural Ecuador is essential for developing targeted interventions aimed at reducing the burden of chronic diseases in this population.

The primary objective of this study was to determine the prevalence of MetS in a rural Ecuadorian population and to assess its association with cardiovascular and endocrine diseases. By identifying key risk factors, this study aimed to inform public health strategies to address the growing burden of chronic diseases in rural Ecuadorian populations.

## 2. Materials and Methods

### 2.1. Study Design

This observational, cross-sectional study was conducted between August and November 2023 in the rural parish of Roberto Astudillo, Milagro, Ecuador. The study design adhered to the Strengthening the Reporting of Observational Studies in Epidemiology (STROBE) guidelines [15]. A cross-sectional design was chosen to evaluate the association between MetS biomarkers (glucose, cholesterol, and triglycerides) and the presence of cardiovascular and endocrine diseases. Ethics approval was granted by the Human Research Ethics Committee of Ecuador (approval number: HCK-CEISH-2022-006). All participants were required to provide written informed consent prior to their inclusion in the study to ensure that they understood the purpose of the research and their rights as participants.

### 2.2. Population and Sample

A total of 200 adults over 18 years of age were recruited from a rural parish using a simple random sampling process. This population was chosen because of its limited access to healthcare services and the higher prevalence of risk factors for chronic diseases related to socioeconomic and environmental factors. Participants were eligible if they were permanent parish residents. Exclusion criteria were pregnancy or any chronic condition that might significantly alter metabolic biomarker levels (e.g., cancer and advanced liver disease). However, participants with and without self-reported or previously diagnosed cardiovascular and endocrine diseases were included, as these conditions were the primary outcomes of interest. This approach allowed us to examine the association between metabolic syndrome components and the risk of developing cardiovascular and endocrine diseases, while ensuring that metabolic measurements were not confounded by unrelated chronic conditions.

The sample size was calculated based on the expected prevalence of metabolic syndrome (MetS) in Latin American populations, which has been reported to range from 20% to 30% in similar settings [16]. Using a 25% estimated prevalence, 95% confidence level, and 5% margin of error, the required minimum sample size was determined to be 288 participants based on standard sample size calculation formulas [17,18]. However, due to logistical constraints and resource availability, we randomly recruited 200 participants from the parish, which still provided sufficient power for detecting associations in our statistical analyses. Although a larger sample size would improve generalizability, our findings remain relevant for understanding the risk of metabolic syndrome in this population.

### 2.3. Data Collection

Data collection was conducted through structured interviews, physical assessments, and laboratory tests following standardized protocols to ensure reliability and validity.

The structured interviews gathered information on demographics (age, gender, education, and income), lifestyle factors (smoking, alcohol consumption, and physical activity), and medical history. To minimize potential bias in self-reported data, a short structured questionnaire was designed to efficiently capture key variables of interest. This questionnaire was tested and validated within the community to ensure its clarity, reliability, and relevance. Additionally, the participants were assured of confidentiality to encourage honest responses.

The laboratory tests involved blood sample collection from all participants after a minimum 12 h fasting period. Glucose, total cholesterol, and triglyceride levels were analyzed using an automated biochemical analyzer in a certified laboratory with internal and external quality control procedures in place to ensure accuracy and reproducibility. These biomarkers were chosen because of their strong association with MetS, cardiovascular diseases, and endocrine disorders [19].

By implementing these standardized procedures and validation steps, we aimed to enhance the accuracy, reliability, and generalizability of our findings.

### 2.4. Variables

The primary outcome variable was the presence of cardiovascular and endocrine diseases, as determined by self-reported physician diagnoses. These include hypertension, coronary artery disease, stroke, diabetes, and other metabolic disorders. The main predictor variables were fasting glucose, total cholesterol, and triglycerides, all of which were treated as continuous variables in the analysis. Metabolic syndrome was defined based on the National Cholesterol Education Program Adult Treatment Panel III (NCEP ATP III) criteria, which require the presence of at least three of the following components: abdominal obesity, high triglycerides, low HDL cholesterol, high blood pressure, and elevated fasting glucose [20]. However, owing to data limitations, HDL cholesterol measurements were unavailable, and we used total cholesterol as a proxy for dyslipidemia. This decision was based on the availability of total cholesterol data in our study and its well-established role as a risk factor for cardiovascular disease. Given these constraints, the prevalence of metabolic syndrome was estimated by identifying participants who met at least three of the following criteria: fasting glucose ≥ 100 mg/dL, triglycerides ≥ 150 mg/dL, and total cholesterol ≥ 200 mg/dL. As a result, we referred to metabolic syndrome indicators rather than full MetS criteria, as we cannot accurately estimate its prevalence with the available data.

Control variables included demographic factors such as age (continuous variable in years), sex (male/female), education level (low: primary or lower; high: secondary or higher), and income level (low: below the national median household income; high: above the median). Behavioral factors included smoking status (yes/no) and alcohol consumption (yes/no). Physical activity was assessed based on self-reported weekly exercise frequency and categorized as low (<150 min/week of moderate-intensity activity), moderate (150–299 min/week of moderate-intensity or 75–149 min/week of vigorous-intensity activity), or high (≥300 min/week of moderate-intensity or ≥150 min/week of vigorous-intensity activity), according to the WHO and AHA guidelines. Additionally, the presence of cardiovascular disease (yes/no; including hypertension, coronary artery disease, and stroke) and endocrine disease (yes/no; including diabetes and other metabolic disorders) was recorded.

### 2.5. Statistical Analysis

All statistical analyses were conducted using R statistical software (version 4.3.2) [21]. Descriptive statistics were used to summarize demographic characteristics and glucose, cholesterol, and triglyceride levels. Continuous variables were expressed as means and standard deviations, while categorical variables were reported as frequencies and percentages.

The Shapiro–Wilk test was used to assess the normality of continuous variables. Non-normally distributed variables were log-transformed for regression analysis. Logistic regression models were used to determine the association between MetS biomarkers and the risk of cardiovascular and endocrine diseases (unadjusted and adjusted for controls). Odds ratios (OR) and 95% confidence intervals (CI) were reported, with significance set at *p* < 0.05.

Model performance was assessed using the area under the receiver operating characteristic curve (AUC), sensitivity, and specificity. Multicollinearity among independent variables was checked using variance inflation factors (VIF), and none of the VIFs exceeded the threshold value of 10.

## 3. Results

### 3.1. Descriptive Statistics

The study included 200 participants with a mean age of 45.6 years (SD = 12.3). Of these, 58% were female and the majority had a low income (73%) and low levels of formal education (62%). Table 1 presents the descriptive statistics for glucose, cholesterol, and triglyceride levels.

The average glucose level was 123.09 mg/dL, indicating that many participants were at a high risk for diabetes, as values above 100 mg/dL suggest impaired fasting glucose or prediabetes. Cholesterol levels were borderline high, with an average of 229.58 mg/dL, exceeding the recommended threshold of <200 mg/dL for optimal cardiovascular health. Triglyceride levels, averaging 188.75 mg/dL, were borderline high but near high-risk levels according to the American Heart Association’s guidelines for cardiovascular health [22].

Based on the available metabolic indicators from Table 1, the estimated prevalence of metabolic syndrome (MetS) in our study population was approximately 40%. This estimate was derived from the proportion of participants who were likely to meet at least three MetS-defining risk factors. The mean fasting glucose level was 123.09 mg/dL, exceeding the metabolic syndrome threshold of ≥100 mg/dL, indicating that a substantial proportion of the participants had hyperglycemia. Similarly, the mean triglyceride level was 188.75 mg/dL, surpassing the threshold of ≥150 mg/dL, suggesting a high prevalence of dyslipidemia. While HDL cholesterol was not directly measured, the high total cholesterol mean of 229.58 mg/dL suggests widespread lipid abnormalities. To estimate the prevalence, we assumed that individuals with glucose ≥ 100 mg/dL, triglyceride ≥ 150 mg/dL, and total cholesterol ≥ 200 mg/dL met the three criteria for MetS. Given that prior studies suggest a substantial overlap among these conditions, we estimated that approximately 40% of the study population met the criteria for metabolic syndrome. However, owing to the absence of direct HDL cholesterol and abdominal obesity measurements, this estimate should be interpreted with caution.

The average age of the participants was 45.6 years (SD = 12.3), and the majority (58%) were female. A significant proportion of the participants had low socioeconomic status, with 73% reporting low income and 62% having low levels of formal education, which are known risk factors for poor health outcomes. In terms of behavioral factors, 34% of participants reported smoking and 40% reported alcohol use, both of which are associated with an increased risk of metabolic and cardiovascular diseases. Physical activity levels were low, with 55% of participants reporting low physical activity, 30% moderate physical activity, and only 15% high physical activity, further increasing the risk of metabolic syndrome and its associated complications.

These descriptive statistics highlight a population with multiple risk factors for cardiovascular and endocrine diseases, including elevated glucose, cholesterol, and triglyceride levels, combined with lifestyle factors such as smoking, alcohol use, and low physical activity. Among the study participants, 28% reported having a cardiovascular disease, while 35% had an endocrine disorder, highlighting the substantial burden of these conditions in the rural population. Given the wide variation in specific conditions, we grouped them into broader categories: cardiovascular diseases, including hypertension, coronary artery disease, and stroke, while endocrine disorders encompassed diabetes, thyroid dysfunction, and other metabolic conditions. This classification allowed for a more structured analysis of their association with metabolic risk factors.

### 3.2. Logistic Regression Analysis

Logistic regression models were developed to assess the relationship between MetS biomarkers and risk of cardiovascular and endocrine diseases. The results are presented in Table 2.

The results of the logistic regression analysis indicated that higher levels of glucose, cholesterol, and triglycerides were significantly associated with an increased likelihood of both cardiovascular and endocrine diseases. For cardiovascular diseases, glucose was the strongest predictor, with each unit increase associated with 8.5% higher odds in the unadjusted model (OR = 1.085) and 6.9% higher odds after adjusting for control variables (OR = 1.069). Similarly, higher cholesterol and triglyceride levels were associated with 1.5% and 2.5% increases in cardiovascular disease odds, respectively, even after adjustment. For endocrine diseases, glucose again had the strongest association, with each unit increasing the odds by 14.5% in the unadjusted model (OR = 1.145) and by 11.8% in the adjusted model (OR = 1.118). Cholesterol and triglyceride levels also showed significant associations with endocrine disease risk, with adjusted odds ratios indicating a 2.9% and 3.4% increase in odds per unit increase, respectively.

### 3.3. Model Performance

The cardiovascular disease model had an AUC of 0.933, indicating excellent discrimination between participants with and without cardiovascular disease. The AUC for the endocrine disease model was even higher (0.993), suggesting a near-perfect discrimination. The sensitivity and specificity were similarly high for both models, with the cardiovascular model achieving a sensitivity of 78.26% and specificity of 86.49%, while the endocrine model achieved 88.89% sensitivity and 95.24% specificity.

## 4. Discussion

The findings of this study indicate that higher levels of glucose, cholesterol, and triglycerides are significantly associated with an increased likelihood of both cardiovascular and endocrine diseases. Among these, glucose was the strongest predictor, with each unit increase associated with 8.5% higher odds of cardiovascular disease in the unadjusted model and 6.9% after adjustment, whereas cholesterol and triglycerides increased cardiovascular disease risk by 1.5% and 2.5%, respectively. For endocrine diseases, glucose remained the most significant predictor, with 14.5% higher odds in the unadjusted model and 11.8% after adjustment, while cholesterol and triglycerides contributed to 2.9% and 3.4% increases in odds, respectively.

These results align with previous research showing that dysregulated glucose metabolism and lipid imbalances are key risk factors for cardiovascular and endocrine diseases, particularly in populations with limited access to healthcare. Furthermore, in rural settings, factors such as high-carbohydrate diets, limited healthcare access, and sedentary lifestyles may exacerbate the effect of hyperglycemia, cholesterol, and triglycerides on disease risk [23,24]. Limited medical interventions and delayed diagnosis in these areas could further contribute to worse health outcomes compared to urban populations. Future studies should explore these contextual influences to develop targeted prevention strategies for rural populations.

The strong association between glucose and endocrine diseases supports the well-established link between hyperglycemia and type 2 diabetes mellitus (T2DM) [25,26]. The persistence of these associations after adjusting for demographic and behavioral factors suggests that these metabolic biomarkers independently contribute to disease risk, reinforcing the need for targeted interventions focused on early detection, lifestyle modifications, and improved healthcare access in rural communities.

### 4.1. Elevated Glucose and Endocrine Disease Risk

The findings of this study reinforce the notion that hyperglycemia is a central component of metabolic syndrome and a direct contributor to endocrine disorders, particularly T2DM. Numerous studies have demonstrated that chronic high blood glucose levels result in insulin resistance, which is a key mechanism in the pathogenesis of T2DM [27,28]. Insulin resistance occurs when cells in the body become less responsive to insulin, resulting in higher blood glucose levels and eventual dysfunction of pancreatic beta cells, which produce insulin [29]. Over time, this leads to full-blown diabetes and a range of associated complications, including microvascular damage, nephropathy, neuropathy, and retinopathy [30,31].

Given the high prevalence of elevated glucose levels in this population, public health interventions aimed at the early detection and management of hyperglycemia are crucial. Screening for elevated fasting blood glucose and glycated hemoglobin (HbA1c) levels can help identify individuals at a risk of developing T2DM. Early intervention, including lifestyle modifications such as dietary changes and increased physical activity, has been shown to effectively prevent or delay the onset of diabetes in high-risk individuals [32,33].

### 4.2. Dyslipidemia and Cardiovascular Disease

Cholesterol and triglycerides were also strongly associated with cardiovascular diseases. This finding supports previous research highlighting the role of dyslipidemia in the development of atherosclerosis and coronary artery disease (CAD). Dyslipidemia, characterized by elevated levels of low-density lipoprotein (LDL) cholesterol and triglycerides as well as low levels of high-density lipoprotein (HDL) cholesterol, contributes to the buildup of fatty plaques in the arteries. This process, known as atherosclerosis, is a major underlying cause of cardiovascular events, such as myocardial infarction (heart attack) and stroke [34,35].

Triglycerides are emerging as independent risk factors for cardiovascular diseases. While elevated LDL cholesterol has traditionally been the primary target for lipid-lowering therapies, recent studies have shown that elevated triglycerides are associated with increased cardiovascular risk, even when LDL cholesterol levels are within the normal range [36,37]. This highlights the importance of comprehensive lipid management in individuals with metabolic syndrome. In this study, the high prevalence of elevated cholesterol and triglyceride levels indicates a significant burden of dyslipidemia in the population, further increasing the risk of cardiovascular events.

### 4.3. Comparison with Regional and Global Studies

Our findings contribute to the understanding of metabolic syndrome (MetS) and its association with cardiovascular and endocrine diseases in a rural Ecuadorian population. Although this study is geographically specific, its relevance extends to other low-resource settings globally, where similar socioeconomic and environmental factors contribute to the burden of metabolic diseases.

The prevalence of MetS observed in our study aligns with findings from other Latin American countries, where MetS rates have been reported to range between 20% and 40%, depending on the population studied [4,10]. In rural settings, limited healthcare access, lower socioeconomic status, and reduced preventive care measures often result in a higher prevalence of late-stage diagnoses of metabolic disorders [15,38]. Similar trends have been documented in Peru and Mexico, where rural populations exhibit elevated metabolic risk factors, despite having a lower prevalence of obesity than urban populations [21].

Our findings are consistent with those of studies from Africa and South Asia, where metabolic syndrome prevalence has been rising due to the increasing adoption of sedentary lifestyles and processed diets [3]. In rural India and sub-Saharan Africa, researchers have noted a transition from infectious to metabolic diseases exacerbated by nutritional shifts, urbanization, and reduced physical activity [6]. The high rates of elevated glucose and dyslipidemia observed in our study further support global evidence that metabolic syndrome is not confined to high-income countries but is increasingly affecting low- and middle-income regions.

Despite these similarities, our study highlights the unique regional factors that may influence metabolic health in Ecuador. The reliance on self-reported diagnoses and limited healthcare infrastructure may result in an underdiagnosis of metabolic and cardiovascular diseases, leading to delayed interventions. Additionally, dietary habits in rural Ecuadorian communities, which often include high carbohydrate and sugar intake, may contribute to the elevated glucose and triglyceride levels observed in our population. These context-specific insights emphasize the need for targeted interventions that consider local dietary patterns, healthcare accessibility, and socioeconomic factors.

### 4.4. The Need for Public Health Interventions

The high prevalence of elevated glucose, cholesterol, and triglyceride levels in this rural Ecuadorian population highlights the urgent need for targeted public health interventions. These findings suggest that a substantial proportion of the population is at high risk for cardiovascular and endocrine diseases due to the presence of metabolic syndrome components.

Lifestyle modifications are the cornerstones of metabolic syndrome management. Improvements in diet and increased physical activity have been shown to be effective in reducing the prevalence of MetS and preventing its associated complications. Dietary interventions that focus on reducing saturated fat intake, increasing fiber consumption, and promoting the intake of whole grains, fruits, and vegetables have been shown to improve lipid profiles and reduce glucose levels [39]. Furthermore, regular physical activity has been shown to improve insulin sensitivity and promote weight loss, which is particularly important in individuals with central obesity, a key component of MetS.

However, lifestyle interventions alone may not be sufficient for individuals at a high risk of cardiovascular and endocrine diseases. Pharmacological interventions, such as statins and metformin, may be necessary to manage elevated lipid and glucose levels. Statins, lipid-lowering drugs, have been shown to significantly reduce the risk of cardiovascular events in individuals with dyslipidemia by lowering LDL cholesterol levels and stabilizing atherosclerotic plaques [40]. Metformin, an insulin-sensitizing drug, has been shown to reduce the risk of developing T2DM, particularly in overweight or obese [41,42].

### 4.5. Barriers to Intervention in Rural Populations

The implementation of effective public health interventions in rural populations presents unique challenges. In rural Ecuador, as in many other low- and middle-income countries, access to healthcare is limited due to geographical barriers, lower healthcare infrastructure, and economic constraints. Rural populations often lack access to regular medical care, screening programs, and medications, all of which are essential for managing metabolic syndromes and preventing complications.

Public health efforts in these regions must focus on improving access to healthcare through mobile clinics, telemedicine, and community health workers who can provide education and screening services. Education campaigns that raise awareness of the risks of MetS and the importance of early detection and lifestyle changes are critical. Additionally, subsidizing medications, such as statins and metformin, could significantly reduce the burden of cardiovascular and endocrine diseases in high-risk populations.

### 4.6. The Role of Socioeconomic Factors

Socioeconomic factors play a crucial role in the prevalence of MetS and its associated risk factors. In this study, a large proportion of participants had low incomes and low levels of formal education. A low socioeconomic status is strongly associated with higher rates of obesity, poor diet, and physical inactivity, all of which are major contributors to the development of metabolic syndrome. Individuals with low incomes may have limited access to healthy food options, relying on inexpensive, processed foods that are high in sugar and unhealthy fats. Additionally, individuals with lower education levels may be less aware of the health risks associated with MetS and less likely to engage in preventive health behaviors [38,43].

Addressing the socioeconomic determinants of health is essential to reduce the prevalence of MetS and its associated complications. Public health policies that aim to improve access to affordable and healthy food options and promote physical activity through community-based programs are needed. Furthermore, educational campaigns that target individuals with lower levels of education can help increase awareness of the importance of managing metabolic risk factors.

### 4.7. Implications for Policy and Practice

The findings of this study have important implications for public health policy and clinical practice in Ecuador and other low- and middle-income countries. Policymakers must prioritize the prevention and management of metabolic syndrome as part of broader efforts to reduce the burden of noncommunicable diseases. This includes investing in the healthcare infrastructure, expanding access to screening programs, and providing subsidies for medications that are critical for managing dyslipidemia and hyperglycemia.

From a clinical perspective, healthcare providers in rural areas should be trained to identify and manage metabolic syndrome. This includes regularly screening patients for risk factors, such as elevated glucose, cholesterol, and triglycerides, and providing appropriate counseling on lifestyle modifications. When necessary, healthcare providers should prescribe pharmacological interventions and ensure that patients have access to the medications that they need to reduce their risk of cardiovascular and endocrine diseases.

Addressing metabolic syndrome (MetS) in rural populations requires personalized approaches tailored to the unique challenges and characteristics of the affected individuals. Personalized healthcare interventions should consider not only metabolic biomarkers, such as glucose, cholesterol, and triglycerides, but also demographic, socioeconomic, and behavioral factors [25]. For instance, integrating community-based health programs with genetic and lifestyle assessments can enhance early detection and targeted management of MetS [42]. Additionally, personalized counseling that aligns dietary recommendations with local food availability and cultural practices can improve adherence to a healthier lifestyles [43]. Mobile health technologies, including wearable devices and telemedicine platforms, can facilitate individualized monitoring and support in remote areas [36]. By leveraging personalized medicine, public health initiatives can achieve greater effectiveness in mitigating the risks of cardiovascular and endocrine diseases associated with MetS in underserved rural communities [34].

### 4.8. Study Limitations

This study has several limitations that should be considered when interpreting the findings. First, the cross-sectional design prevented the establishment of causal relationships between metabolic syndrome (MetS) biomarkers and the risk of cardiovascular and endocrine diseases. Second, reliance on self-reported cardiovascular and endocrine disease diagnoses introduces the potential for recall bias or underreporting, particularly in a rural population with limited access to healthcare. However, by excluding participants with previously confirmed diagnoses, we ensured that our study population was representative of at-risk individuals rather than those already diagnosed and under treatment.

Additionally, the sample size of 200 participants, which is sufficient to detect associations, may limit the generalizability of our findings to other rural or urban populations in Ecuador and beyond. Another limitation is the absence of dietary and genetic data, which could provide further insights into personalized risk factors and interventions. Furthermore, owing to data constraints, metabolic syndrome prevalence was estimated using available metabolic indicators, such as fasting glucose, triglycerides, and total cholesterol, rather than the standard NCEP ATP III criteria, which include HDL cholesterol and abdominal obesity. While total cholesterol was used as a proxy for dyslipidemia, and our estimates align with those of prior studies, the lack of direct HDL cholesterol and obesity measurements may impact the accuracy of our prevalence estimation.

Moreover, although additional variables could have further enriched the study, data collection was constrained by extremely limited funding. In future research, with increased resources, we aim to expand the study scope by ideally conducting a census of the entire population to obtain a more comprehensive assessment. Finally, while we propose personalized medicine solutions, their implementation and effectiveness in this population remain to be evaluated through future longitudinal or intervention studies.

### 4.9. Future Research Directions

Although this study provides important insights into the prevalence of MetS and its association with chronic diseases in a rural Ecuadorian population, further research is needed to fully understand the impact of MetS in this context. Longitudinal studies that track the progression of MetS over time and its relationship with cardiovascular and endocrine diseases would provide valuable information regarding the natural history of MetS and its complications. Additionally, research exploring the genetic and environmental factors contributing to MetS in rural populations could help identify high-risk individuals and inform targeted interventions.

There is also a need for research evaluating the effectiveness of public health interventions in reducing the prevalence of MetS in rural areas. Intervention studies that assess the impact of dietary changes, physical activity programs, and pharmacological treatments on MetS outcomes would provide valuable evidence to guide public health strategies.

## 5. Conclusions

This study highlights the burden of metabolic syndrome in the rural Ecuadorian population and its association with cardiovascular and endocrine diseases. The high prevalence of metabolic risk factors reinforces the need for targeted public health interventions, including screening programs, such as community-based health screenings and mobile clinics. The use of HbA1c as a diagnostic tool offers advantages over fasting glucose by providing a long-term measure of glycemic control and reducing variability. Dyslipidemia, particularly elevated LDL cholesterol and triglyceride levels, is a significant contributor to coronary artery disease (CAD), emphasizing the need for lipid management strategies. Dietary interventions should prioritize a high fiber intake, reduced refined carbohydrates, and healthy fats, while physical activity, such as daily moderate-intensity aerobic exercise and resistance training, can improve insulin sensitivity and lipid metabolism.

Addressing healthcare access disparities, improving early detection, and implementing targeted lifestyle modifications are essential for reducing the burden of metabolic syndrome in underserved communities. Future research should explore the long-term impact of metabolic syndrome on disease progression through longitudinal studies, assess the effectiveness of tailored interventions over time, and incorporate additional metabolic markers, including HDL cholesterol and abdominal obesity, to refine prevalence estimates and enhance comparability with global studies. Expanding this research to similar rural populations will help generalize findings and support broader public health strategies.

## Figures and Tables

**Table 1:** Summary of participant demographics and health indicators.

Variable	Mean (SD)/%	Min	Max	Skewness	Kurtosis
Glucose (mg/dL)	123.09 (49.49)	71.3	386.1	2.6	8.96
Cholesterol (mg/dL)	229.58 (56.92)	90.9	412.2	0.2	0.25
Triglycerides (mg/dL)	188.75 (62.97)	90.3	356.2	0.58	−0.11
Age (years)	45.6 (12.3)	18	80	-	-
Gender (% Female)	58%	-	-	-	-
Education Level (% Low)	62%	-	-	-	-
Income Level (% Low)	73%	-	-	-	-
Smoking (% Yes)	34%	-	-	-	-
Alcohol Use (% Yes)	40%	-	-	-	-
Physical Activity (% Low/Moderate/High)	55%/30%/15%	-	-	-	-
Cardiovascular Disease (% Yes)	28%	-	-	-	-
Endocrine Disease (% Yes)	35%	-	-	-	-

**Table 2:** Logistic regression analysis of metabolic syndrome biomarkers for cardiovascular and endocrine disease risk (unadjusted and adjusted for control variables).

Predictor	Outcome	Unadjusted OR (95% CI)	*p*-Value (Unadj.)	Adjusted OR (95% CI)	*p*-Value (Adj.)
Glucose	Cardiovascular	1.085 (1.057, 1.122)	<0.001	1.069 (1.031, 1.099)	<0.001
Cholesterol	Cardiovascular	1.017 (1.010, 1.025)	0.02	1.015 (1.006, 1.024)	0.013
Triglycerides	Cardiovascular	1.031 (1.020, 1.046)	<0.001	1.025 (1.013, 1.037)	<0.001
Glucose	Endocrine	1.145 (1.099, 1.192)	<0.001	1.118 (1.075, 1.164)	<0.001
Cholesterol	Endocrine	1.038 (1.020, 1.051)	<0.001	1.029 (1.018, 1.040)	<0.001
Triglycerides	Endocrine	1.041 (1.025, 1.056)	<0.001	1.034 (1.024, 1.044)	<0.001

## Data Availability

The raw data supporting the conclusions of this article will be made available by the authors on request.
